# A spontaneous hematoma arising within an intrapancreatic accessory spleen: A case report and literature review

**DOI:** 10.1097/MD.0000000000008092

**Published:** 2017-10-13

**Authors:** Guo-Dong Shan, Wen-Guo Chen, Feng-Ling Hu, Li-Hua Chen, Jing-Hua Yu, Hua-Tuo Zhu, Qi-Qi Gao, Guo-Qiang Xu

**Affiliations:** a Department of Gastroenterology; b Department of Pathology, the First Affiliated Hospital, College of Medicine, Zhejiang University, Hangzhou, China.

**Keywords:** accessory spleen, CT, hematoma, intrapancreatic, MRI

## Abstract

**Rational:**

Hematoma arising within an intrapancreatic accessory spleen (IPAS) is an extremely rare pathological entity.

**Patient concern:**

We present the case of a 39-year-old man with acute abdominal pain.

**Diagnoses:**

The patient was initially diagnosed as pancreatic cystic neoplasm according to CT and MRI imaging.

**Interventions:**

Distal pancreatectomy was conducted because of the possibility of malignancy.

**Outcomes:**

Surgical resection showed that the lesion was a hematoma in an IPAS.

**Lessons:**

Our case indicated that the differential diagnosis of hematoma in IPAS should be born in mind for cases with cystic neoplasm in tail of pancreas and an epidermoid cyst arising within an intrapancreatic accessory spleen (ECIAS).

## 1 Introduction

An intrapancreatic accessory spleen (IPAS) is a relatively rare clinical presentation, with the prevalence of 1.7% in general population.^[1]^ A hematoma arising within an IPAS is an extremely rare pathological entity. To our knowledge, no hematoma in an IPAS has been reported previously in English literature. We herein report a case of a spontaneous hematoma arising in an IPAS that was preoperatively diagnosed as a mucinous cystic neoplasm; and resected using distal pancreatectomy.

## 2 Case presentation

A 39 years old male presented with a 3-day history of epigastric pain. The patient had no comorbidities and was not on any medications at time of presentation. He had normal vital signs, and abdominal examination, except for mild epigastric tenderness. Consent to conduct of the case report was obtained from the Ethics Committee of the First Affiliated Hospital of Zhejiang University.

**Figure 1:**
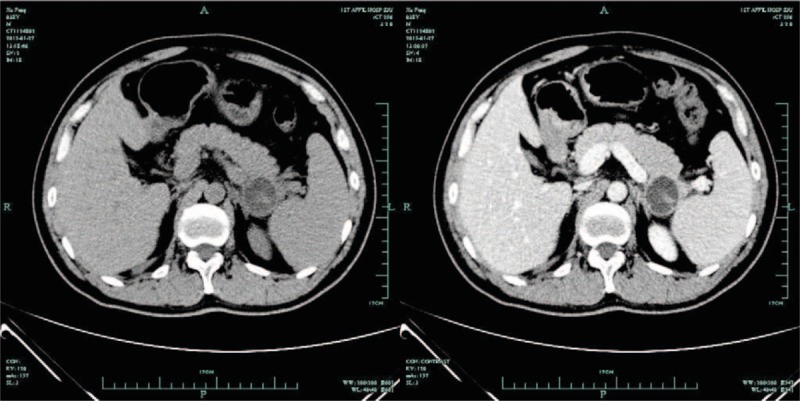
CT scans showed a cystic lesion in tail of pancreas.

**Figure 2:**
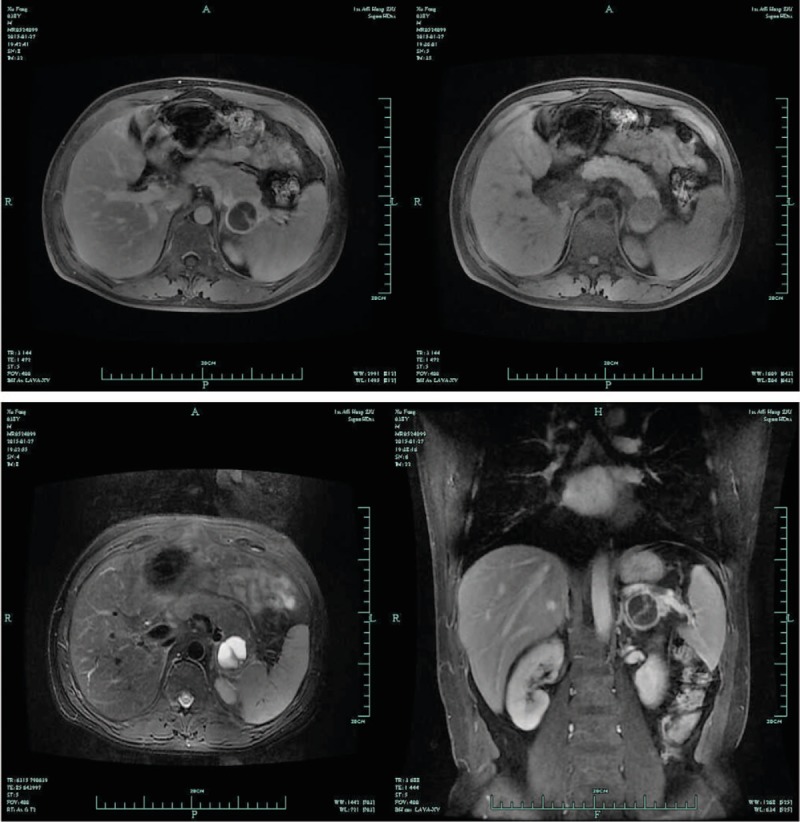
A cystic lesion in tail of pancreas presented lower intensity on T1-weighed MRI and higher intensity on T2 MRI study.

Laboratory tests showed normal liver and kidney functions. The amylase, lipase, Tumor markers and coagulation levels were all normal. Tumor markers and coagulation levels were all normal. In order to find out the cause of abdominal pain, the patient received abdominal computerized tomography (CT) and magnetic resonance imaging (MRI) scan. The results showed that a well-defined cystic neoplasm without enhancing mural nodes (3.3 cm × 3.1 cm in size), was located in the tail of pancreas and approaching to splenic hilus (Figs. 1 and 2). Distal pancreatectomy was conducted because of the possibility of malignancy.

**Figure 3:**
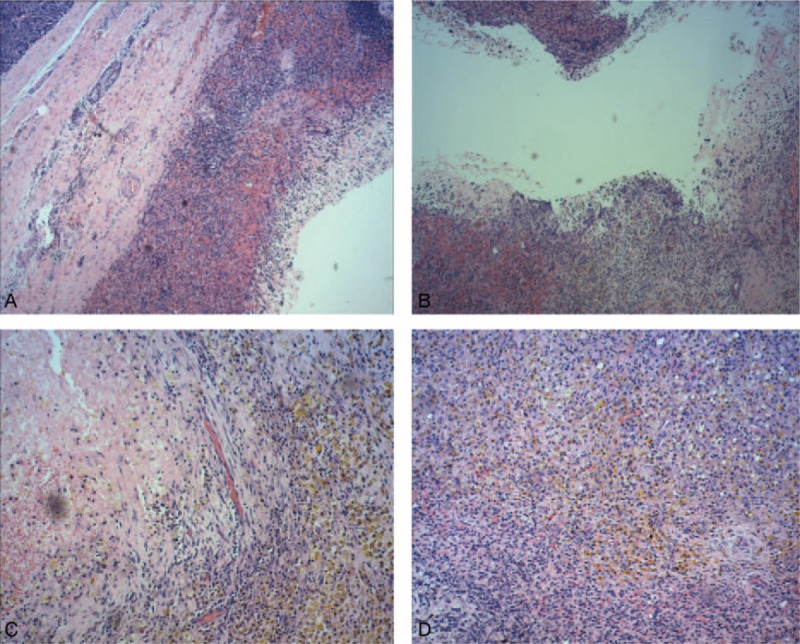
(A and B) Old hemorrhage in spleen tissue. (C and D) Old hemorrhage in spleen tissue and formation of capsule wall, surrounded by pancreatic tissue.

The surgical specimen contained a well-demarcated cyst cavity, that was 3.5 cm × 2.5 cm in size, and surrounded by pancreatic tissue on macroscopic appearance. Microscopically, the capsule wall was surrounded by spleen tissue, containing hemorrhage, hemosiderosis, chronic inflammatory cells, and histiocyte collection (Fig. 3A–D). Therefore, a hemotoma in an IPAS was confirmed by pathologic diagnosis. The patient recovered from surgery without complications and was discharged 6 days later. Six months after operation, the patient was followed up and no discomfort was found. Meanwhile, laboratory examinations including blood routine examination, amylase, and biochemical tests were normal. Abdominal ultrasound was also confirmed as normal.

## 3 Discussion

A hematoma in an accessory spleen was reported in the past.^[2]^ But a hematoma in an IPAS has not been reported hitherto. There are many causes for abdominal hemotoma, including: trauma, coagulopathies, postsurgical conditions, or interventional medical procedures performed either for diagnosis or therapeutic purposes.^[3]^ In this case, according to all kinds of examination results and past medical history, the common causes were all excluded. Therefore, a spontaneous hemotoma in an IPAS was considered.

For the diagnostic modalities of a hemotoma in abdomen, the noninvasive diagnosis primarily depends on CT and MRI features. The signals of the abdominal hematoma on both CT and MRI were changed with the passage of time. The abdominal hematomas on unenhanced CT imaging are typically hyperdense (40–60 HU) in the acute phase and hypodense in the chronic phase, due to time-related changes in hemoglobin levels.^[4]^ MRI is more sensitive than CT in the diagnosis of abdominal hematomas. Usually the hematoma presents as high signal intensity on T1-weighted images. The signal within the lesion on MRI also can vary with the passage of time.^[5]^ But in our case, the density of hematoma (8–10 HU) is similar with cystic fluid on unenhanced CT imaging and no high signal intensity on T1-weighted images was found. We guess that the lesion might be a chronic hematoma, although the patient presented the symptom of acute pain. On the imaging, a hematoma in IPAS mimics a cyst neoplasm in the tail of pancreas and an epidermoid cyst arising within an intrapancreatic accessory spleen (ECIAS). ECIAS is very uncommon. There was no more than 40 cases reported previously in English literature.^[6–10]^ Therefore, the patient was misdiagnosed as pancreatic cystadenoma at first.

Endoscopic ultrasound (EUS) is a key modality for the evaluation of suspected pancreatic lesion, as the entire pancreatic gland can be demonstrated with high spatial resolution from the stomach and duodenum. Under EUS, a hematoma and a cyst lesion has the same image which presents as hypoechoic fluid collection with or without internal separations. It is difficult to differentiate a hematoma in IPAS from ECIAS and cyst neoplasm in the tail of pancreas. For a pancreatic cyst lesion, in order to avoid unnecessary surgical intervention, it is necessary to sample the cyst contents for cytologic and fluid analysis by endoscopic ultrasound guided fine needle aspiration (EUS-FNA). For the chronic hemotoma, Ulla-Rocha et al^[11]^ reported that 1 case of perigastric hematoma and 1 case of perirectal hematoma were treated successfully with EUS-guided drainage and stent placement. Therefore, it is safe to perform EUS-FNA procedures for a chronic hematoma. In this case, the patient refused to take EUS-FNA procedures and received surgery directly.

In conclusion, a hematoma arising in IPAS is a rare entity that can mimic cystic lesions in the tail of pancreas and ECIAS. Our case indicted that the differential diagnosis of hematoma in IPAS should be born in mind for cases with cystic neoplasm in tail of pancreas. In order to avoid unnecessary surgical intervention, cytologic and fluid analysis of lesion by EUS-FNA in chronic hematoma phase is recommended.
